# An Observational Study on Chronic Pain Biomarkers in Fibromyalgia and Osteoarthritis Patients: Which Role for Mu Opioid Receptor’s Expression on NK Cells?

**DOI:** 10.3390/biomedicines11030931

**Published:** 2023-03-17

**Authors:** Valentina Malafoglia, Sara Ilari, Chiara Gioia, Laura Vitiello, Michael Tenti, Cristina Iannuccelli, Costanza Maria Cristiani, Cinzia Garofalo, Lucia Carmela Passacatini, Giuseppe Viglietto, Antonio Sili Scavalli, Carlo Tomino, Vincenzo Mollace, William Raffaeli, Manuela Di Franco, Carolina Muscoli

**Affiliations:** 1 Fondazione ISAL, Institute for Research on Pain, 47921 Rimini, Italy; 2 Institute of Research for Food Safety & Health (IRC_FSH), Department of Health Sciences, University ‘Magna Graecia’ of Catanzaro, 88100 Catanzaro, Italy; 3 Rheumatology Unit, Department of Internal Medicine and Medical Specialties, Sapienza University of Rome, 00161 Rome, Italy; 4 Laboratory of Flow Cytometry IRCCS San Raffaele Roma, 00166 Rome, Italy; 5 Department of Experimental and Clinical Medicine, University ‘Magna Graecia’ of Catanzaro, 88100 Catanzaro, Italy; 6 Scientific Direction, IRCCS San Raffaele Roma, 00166 Rome, Italy

**Keywords:** Mu-Lympho-Marker (MLM), Mu opioid receptor, chronic pain diagnosis, chronic pain biomarkers, pain biomarkers, fibromyalgia biomarkers, osteoarthritis biomarkers, opioid receptors, B cells, natural killer cells

## Abstract

The evaluation of chronic pain is challenging because of the lack of specific biomarkers. We identified the Mu opioid receptor-positive (Mu+) B cell percentage of expression, named Mu-Lympho-Marker (MLM), as a candidate marker for chronic pain in fibromyalgia (FM) and osteoarthritis (OA) patients. Here, we investigate the role of MLM on natural killer (NK) cells in the same patients. Twenty-nine FM and twelve OA patients were analyzed, and twenty-three pain-free subjects were considered as the control group. Blood samples were collected to perform immunophenotyping and Western blot analysis. Biological and clinical data were statistically analyzed. The final results showed that the percentage of NK cells expressing Mu was statistically lower in FM and OA patients than in pain-free subjects, as already demonstrated for B cells. A Western blot analysis was performed in order to detect NK cells’ functional status. Moreover, the correlation analysis of MLM expression with pharmacological therapy did not show any significant results. In conclusion, here, we confirm the role of MLM as a suitable marker for chronic pain and underline NK cells as a new possible immune cell type involved in the “Mu opioid receptor reserve theory”.

## 1. Introduction

Chronic pain represents a significant social, clinical, and economic problem in communities worldwide. In recent years, there has been an enormous interest in improving the diagnostic processes for pain, in order to meliorate the therapeutic approach [1]. However, the most intriguing aspect in pain diagnosis is the subjectivity of a single patient to elaborate and describe their own suffering, considering both pain sensory and affective characteristics. That is why, in 2020, the official definition of pain as “an unpleasant sensory and emotional experience associated with actual or potential tissue damage, or described in terms of such damage” [2] has been improved with the removal of the word “described”, considering, sometimes, it is impossible to explain a painful experience with words [3].

More than in other scientific scenarios, much attention is paid on biomarker identification in the pain field. The need of an objective and unequivocal tool for pain diagnosis or prognosis is pivotal. In definition, a biomarker is an indicator of a biological or pathological process or even a symbol of a pharmacological response [4]. It is usually considered a characteristic stable over the time, in physiological condition, and easy to detect with low costs [5]. Thus, one of the goals for a good diagnostic approach is definitely to find peripheral biomarkers through a simple and quick blood sample [6]. In the field of pain research, there is a great interest in the peripheral detection of markers because of the hypothesis that hyperalgesia is often considered as the result of an interplay between nervous and immune systems. The connection between the two systems lies in opioid receptors expressed both peripherally and centrally in nervous cells, immune cells, and tissues [7]. Thus, in the last few years, we have studied the role of Mu opioid receptor’s expression on immune cells as a possible diagnostic biomarker for chronic pain. In particular, we identified the Mu+ B cell percentage, named the Mu-Lympho-Marker (MLM), as a biomarker in fibromyalgia (FM) and osteoarthritis (OA) pain patients [8]. We reported that, starting from a blood sample, the MLM allows identifying differences between patients with a diverse intensity of pain. In detail, we showed that chronic pain-suffering patients present a lower percentage of Mu+ B cells with respect to pain-free subjects. Starting from these findings, we postulated a deficit of Mu opioid receptor “reserves” on B cells, which leads to an “altered endogenous opioid analgesic activity” and, as a consequence, to pain development.

As other immune cells, natural killer (NK) cells express opioid receptors on their surfaces. It is well known that NK cells play a central role in innate immunity, as they mediate early defenses through innate cytotoxicity against microbial-infected cells and malignant-transformed cells [9,10,11,12]. NK cells comprise ten percent of circulating lymphocytes in the peripheral blood, and they are, almost in total, CD56dim CD16bright cells. They carry out cytotoxic functions via direct or indirect cell identification. The direct recognition depends on NK cell receptors interactions with inhibiting or activation ligands presented on nucleated cells. The activating ligands are constitutively expressed or are induced by stress, infections, or malignant transformations. The main inhibitory ligands are the human leukocyte antigen (HLA) class I molecules. When NK cell receptors recognize the inhibitory ligand of a normal cell, then an inhibitory signal is transmitted, blocking the lysis of the target cell [13].

The indirect pathway consists of antibody-dependent cytotoxicity involving the CD16 receptor. Moreover, the NK killing mechanism of target cells can occur by both the exocytosis of granules, containing cytotoxic enzymes, or by receptor-mediated apoptosis [10].

In the context of pain research, several studies focused their attention on Mu opioid receptors expressed on NK cell surfaces [14,15]. In the past few decades, elevated Mu opioid receptor mRNA levels have been shown to be associated with a low percentage of NK cells in noncancer pain patients treated for a long time with intrathecal morphine [16]. Accordingly, the authors demonstrated that morphine-induced NK cell suppression was abolished in Mu-deficient mice [17]. However, the effect of opioid stimulation on NK cell functions and phenotype expression are still debated. On the one hand, opioid-induced suppression of NK cytotoxic function in patients with acute and chronic pain has been extensively described [15,18]. This effect was attributed to opioid signaling through both classical opioid receptors and toll-like receptor 4 (TLR4) [14,19] and could be reverted by the use of naloxone, an opioid receptor antagonist [20]. On the other hand, several studies indicated that opioid administration did not alter NK cell activity and reported a normal expression of NK cell-activating molecules and -triggering receptors [21,22]. Moreover, the evidence suggested that the immunomodulatory effect might depend on the type of opioids used [23]. Therefore, further investigations are needed to elucidate the role of opioids in the regulation of NK cell functions and the meaning of opioid receptors on the immune cell surface. This intriguing interplay across endogenous antinociceptive and immune systems, in addition to the recent findings about the MLM expression on B cells in FM and OA patients, has encouraged us to investigate the role of Mu opioid receptors on NK cells in the context of our “Mu opioid receptor reserve theory”. Thus, in the proposing work, we further studied the role of MLM expression on NK cells, aiming to identify a new immune cell population involved in the network of pain biomarkers. Here, we analyzed the percentage of expression of Mu+ NK cells in order to detect differences across FM, OA pain patients, and pain-free subjects. Moreover, we detected NK cells’ functional status through a Western blotting analysis. We also focused our attention on pharmacological therapy for each patient in order to find any correlation with the MLM expression.

## 2. Materials and Methods

### 2.1. Trial Design

This is an observational, cross-sectional single-blinded diagnostic trial. The protocol has been designed by the ISAL Foundation, the IRC-FSH, Department of Health Science of University of Catanzaro, the Rheumatology Unit of Sapienza—University of Rome, and IRCCS San Raffaele Roma (Rome). The current study is approved by the institutional independent ethics committee of Sapienza University of Rome, with the name of “I markers Bio-Psico-Sociali nella sindrome fibromialgica” (Fibromyalgia syndrome Bio-Psycho-Social markers), on 8 March 2018 (Ref. 4937), and the trial is registered in the ISRCTN registry, ID: ISRCTN24645566, 10 December 2018.

### 2.2. Participants

All the patients enrolled were female. Twenty-nine (29) FM patients, twelve (12) OA patients, and twenty-three (23) pain-free healthy people (Ctrl (-)) were analyzed. FM patients, following both the 1990 and 2010 ACR criteria [1,2], referred to the Clinic for the Diagnosis and Therapy of Fibromyalgia, Rheumatology Unit, Sapienza University of Rome (AOU Policlinico Umberto I, Rome). OA patients and pain-free subjects referred to the Rheumatology Unit, Department of Internal Medicine and Medical Specialties, Sapienza University of Rome. For all the patients, ranking between 18 and 65 years old, pharmacological therapy was registered, and patients currently assuming opioid formulations were excluded, considering that opioid receptor expression and activity could be influenced by opioids. Patients with rheumatic pathologies were also excluded. During the first clinical examination, all the participants signed a specific informed and consent form and General Data Protection Regulation (GDPR) obligations form. After the inclusion/exclusion criteria analysis and clinical evaluation, participants were included in a sequential numeric code list. Only the medical doctor could access patient names in order to protect confidentiality.

### 2.3. Clinical Measurements

Clinicians of Rheumatology Unit, Sapienza University of Rome, assessed the clinical characteristics. The day of enrolment, clinical data were collected in a specific CFR following the numerical code for single patients. The pain score was reported using the Numerical Rating Scale (NRS), where zero represents “no pain” and ten “the worst possible pain”.

### 2.4. Blood Collection

Fifteen milliliters of peripheral blood were collected during the clinical examination in order to be analyzed within the next 24 h. Research biologists, receiving samples with the numerical code, were blind to the patients’ personal information and therapy.

### 2.5. Immunophenotyping Analysis

Peripheral blood samples were stained with the following antibodies: APC-conjugated anti-Mu (LSBio, Milan, Italy), BUV395-conjugated anti-CD45 (Becton Dickinson—BD, Florence, Italy), PE-Cy7-conjugated anti-CD16 (BD), BB515-conjugated anti-CD-56 (BD), and BV480-conjugated anti-CD3 (BD) for 20 min at 4 °C. Stained samples were incubated at room temperature for 15 min with BD FACS Lysing Solution (BD) and then centrifuged for 5 min at 1500 RPM. After washing, samples were suspended in PBS and then acquired on a LSR Fortessa X20 flow cytometer (BD). Analyses were performed using Diva software in order to detect the percentage of Mu+ NK cells in FM and OA patients and the pain-free control group.

### 2.6. Lymphocytes Isolation

Peripheral blood mononuclear cells (PBMCs) were isolated using a Ficoll (GE Healthcare, Milan, Italy) density centrifugation gradient. Fifteen milliliters of whole blood sample were diluted with PBS (1:1 dilution) and carefully layered over a Ficoll medium. Layered whole blood was centrifuged at 2200 RPM for 20 min at 20 °C at the acceleration max without a break. The resulting PBMCs were washed with PBS and centrifuged at 1800 RPM for 5 min at the acceleration and deceleration max; then, the pellet was collected, suspended with PBS, and centrifuged at 800 PRM for 10 min (acceleration max, deceleration 4). The obtained PBMCs were used for protein extraction and the Western blot analysis.

### 2.7. Proteins Extraction

PBMCs were homogenized with lysis buffer (20 mM Tris-Base, 150 mM, NaCl, 10% Glycerol, 2 mM EDTA, 1% CHAPS, and 1% protease inhibitor cocktail) in a 1:3 *w*/*v* ratio. Solubilized extracts were centrifuged at 14,000 rpm for 20 min at 4 °C. The resulting supernatants were used for immunoprecipitation and the Western blot analysis to evaluate NK cell activity.

### 2.8. Western Blot Analysis

For immunoprecipitation, 50 μL of protein A–sepharose 4B (Sigma, Milan, Italy) resin was washed four times in PBS. In each wash, the resin was mixed with fresh PBS and collected after centrifugation at 14,000 rpm at 4 °C for 1 min. The cleaned resin was suspended in lysis buffer, coated with anti-phosphotyrosine antibodies (Biolegend, San Diego, United States), and mixed overnight at 4 °C. The beads were washed four times in PBS and added to each 300 μg of homogenate cell supernatant, then incubated overnight at 4 °C. After sample centrifugation at 14,000 rpm for 20 min at 4 °C, each pellet was collected. The protein concentration was determined using the Pierce BCA protein assay (Thermo scientific, Rockford, USA). Immunoprecipitated proteins were resolved in 10% SDS-PAGE mini-gels and transferred to nitrocellulose membranes. Membranes were blocked for 1 h at room temperature in 1% BSA/0.1% thimerosal in 50 mM Tris-HCl (pH 7.4)/150 mM NaCl/0.01% Tween-20 (TBS/T). After blocking, membranes were incubated overnight at 4 °C with anti-KIR (1:1000; Bio-Rad, Segrate (Milan) Italy) and anti-actin (1:5000; Sigma, Milan, Italy) antibodies. Membranes were then washed with TBST and incubated with secondary monoclonal antibodies (anti-mouse 1:10,000 GE Healthcare, Milan, Italy) conjugated to horseradish peroxidase for one hour at room temperature. After washes, the proteins were visualized by enhanced chemiluminescence (ECL; GE Healthcare). No differences were observed between actin lanes. The quantification of the protein bands of interest was determined by densitometry using Molecular Dynamics ImageQuant 5.2 software (GE Healthcare, Milan, Italy).

### 2.9. Statistical Analysis

#### 2.9.1. Statistical Analysis for Immunophenotyping

We used one-way ANOVA both for group comparisons and for intra-group homogeneity assessments. All data were expressed as the mean ± S.E.M. Tukey’s least significant difference multiple comparisons was used for the post hoc analysis following one-way ANOVA to compute the probability values (*p*) in the two-group comparison. A *p* threshold of 0.05 was considered for statistical significance.

#### 2.9.2. Statistical Analysis for Clinical Correlations Evaluation

The frequency of Mu+ NK cells was evaluated for its association with biological and/or clinical variables by using GraphPad Prism version 5. For continuous variables (NRS and age), linear regression was used. The nonparametric analysis, corrected for multiple comparisons using Dunn’s test, was performed for the comparisons between therapy before enrollment and Mu+ NK cell percentage. For all the analyses, *p*-value < 0.05 was considered as significant.

## 3. Results

### 3.1. Patients’ Characteristics

**Figure 1:**
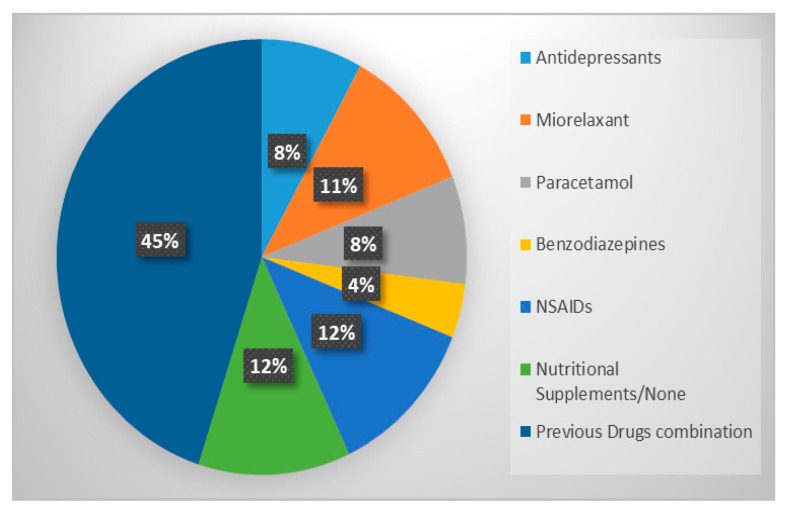
Drugs before enrollment.

Patients’ clinical data were collected in a specific Case Report Form (CRF) based on the exclusion and inclusion criteria (see Section 2.2). The CRF includes the gender and age of patients, clinical characteristics (Table 1), and pharmacological history (Figure 1). The 11-point Numerical Rating Scale (NRS) was used to measure the pain intensity (where 0 = no pain and 10 = worst possible pain) [24].

The totality of the enrolled patients were female, mean ages 54 ± 8.8; 45% were FM patients (*n* = 29), 19% were OA patients (*n* = 12), and 36% (*n* = 23) were pain-free people (Ctrl (-)) (Table 1).

All the patients were following a pharmacological therapy, stable for three months before enrollment: 8% antidepressants, 11% myorelaxants, 8% paracetamol, 4% benzodiazepines, 12% NSAIDs, 12% nutritional supplements and 45% previous drug combinations (Figure 1). None used opioids for pain treatment (see exclusion criteria, Section 2.2).

All of the FM and OA patients ranked between moderate and severe NRS scores (NRS FM patients: mean = 8; NRS OA patients: mean = 5).

### 3.2. Biological Results

#### 3.2.1. Immunophenotyping Analysis: Mu+ NK Cells Percentage of Expression

**Figure 2:**
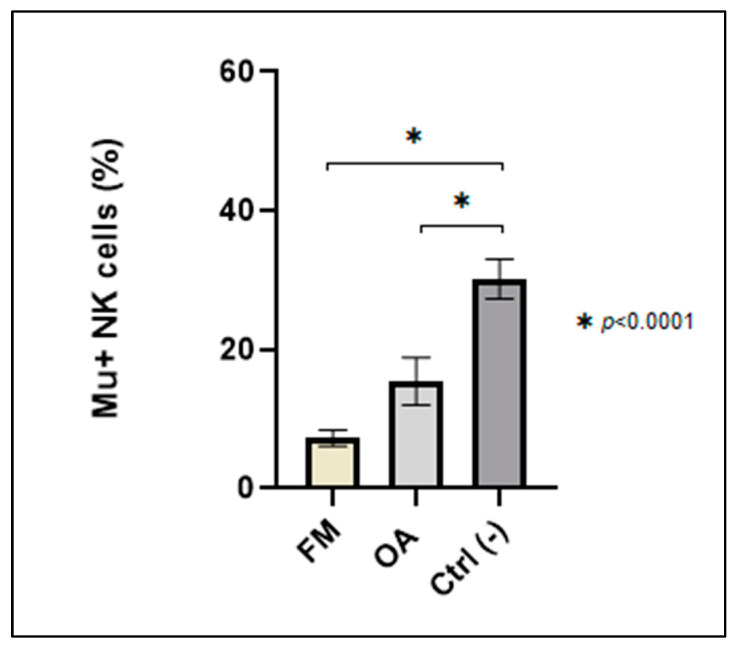
Flow cytometry analysis of Mu+ NK cells in FM and OA patients and Ctrl (-) subjects. The percentage of expression of Mu+ NK was significantly lower in FM and OA patients with respect to the Ctrl (-) (*p* < 0.0001).

Blood samples were analyzed through flow cytometry in order to detect the percentage of expression of Mu+ NK cells. We found intra-group homogeneity (FM mean: 7.3 ± 1.2; OA mean: 15.46 ± 3.4; Ctrl (-) mean: 30.18 ± 2.8), and we registered differences in the percentage of expression of Mu+ NK cells between Ctrl (-) and FM (*p* < 0.0001) and between Ctrl (-) and OA (*p* < 0.0001) (Figure 2).

#### 3.2.2. Western Blot Analysis

NK cells are directly involved in immune diseases, participating in immune responses with innate cytotoxicity, cytokines production, and crosstalk activity with dendritic cells and lymphocytes. The study of HLA class I has been crucial to understanding NK cell functions [9,10,11]. Here, we focus our attention on killer cell immunoglobulin-like receptor (KIR), key regulator of NK cells, and a specific inhibitor receptor, which recognizes classical HLA class I molecules. The KIR family is encoded by 14 polymorphic genes, which can transduce activating or inhibitory signals [18]. In particular, the NK inhibitory signaling cascade initiates with the phosphorylation of the ITIM (inhibitory motif based on tyrosine immunoreceptors) domain [25]. For this reason, we analyzed ITIM phosphorylation through the Western blotting analysis in order to evaluate the status of the activation of NK cells.

**Figure 3:**
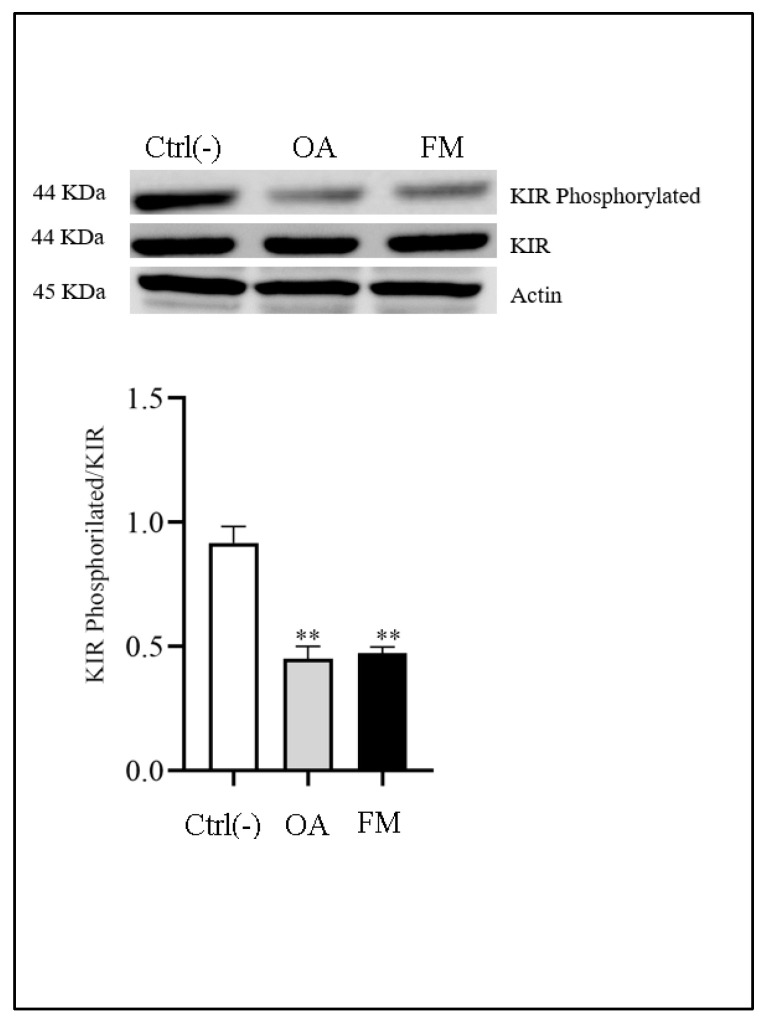
KIR expression on NK cells in FM and OA patients and the Ctrl (-). KIR and KIR phosphorylated were first normalized with actin, and then, these values were used to obtain a phosphorylated/total KIR ratio. The results are expressed as the mean ± SEM for 6 patients. ** *p* < 0.005 vs. Ctrl (-).

Our preliminary data show a significant reduction in expression of the KIR phosphorylation domains in OA and FM patients compared to the Ctrl (-). Probably, these events could lead to a different functionality/activation of KIR, which would compromise NK cell functions (Figure 3).

### 3.3. Mu+ NK Cells Expression and Correlations Analysis

#### 3.3.1. Correlation between Intensity of Pain (NRS Scale) and Mu+ NK Cells Percentage of Expression

**Figure 4:**
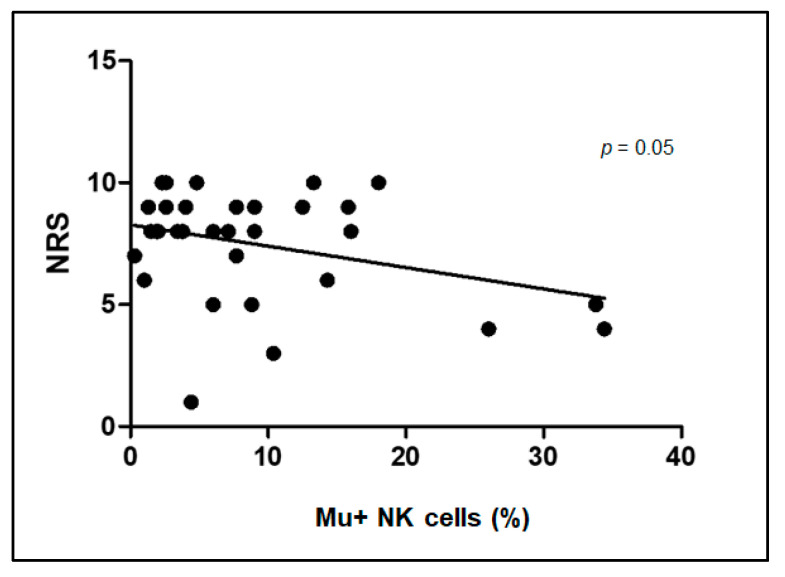
Trend of negative correlation between Mu+ NK cell percentage and NRS values. Linear regression: R square = 0.11; *p* = 0.05.

Following our previous published data [1] showing differences in the percentage of the expression of Mu+ B lymphocytes between FM and OA severe/moderate pain patients and Ctrl (-) subjects, we wanted to detect whether such differences were also significant considering Mu+ NK cell percentages. Thus, we analyzed together FM and OA patients, correlating Mu+ NK cell percentages of expression with NRS values. Figure 4 shows the trend of Mu+ NK cell percentages to negatively correlate with the NRS values (*p* value = 0.05). At a higher level of Mu+ NK cell percentage is a correspondingly lower level of NRS.

#### 3.3.2. Comparison between Therapy before Enrollment and Mu+ NK Cells Percentage of Expression

Considering pain as the main disabling symptom for FM and OA patients, all the participants at the trial followed a pharmacological pain therapy before their enrollment.

**Figure 5:**
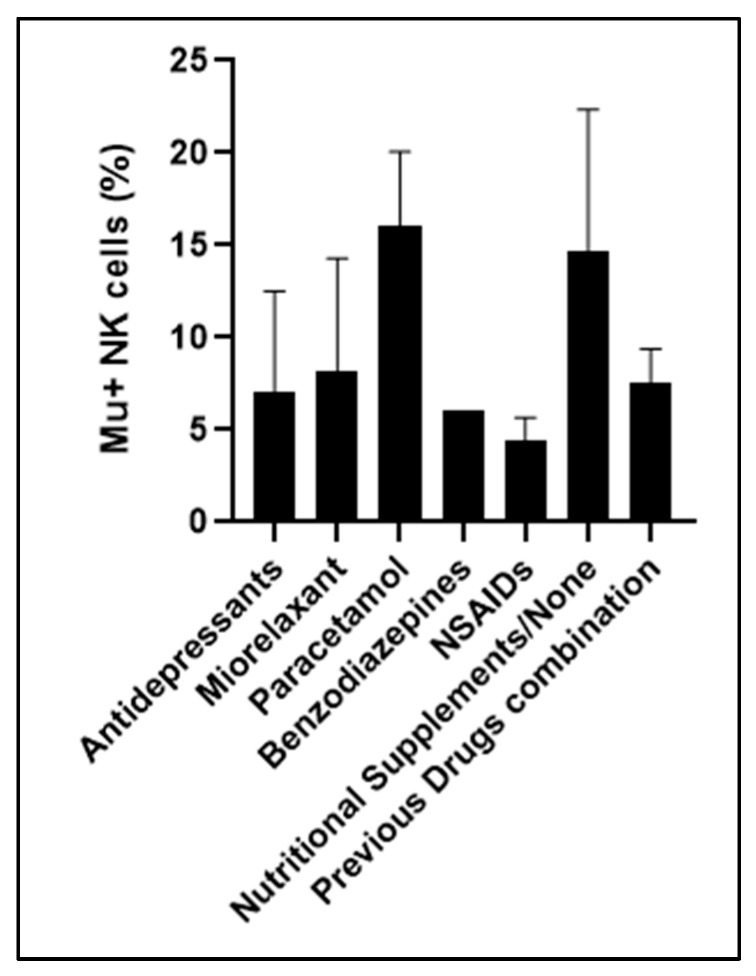
Drug therapies before enrollment, by considering all the patients in their totality, in comparison with Mu+ NK cell percentages. The therapy did not influence the Mu opioid receptor expression.

OA patients, who usually suffer inflammatory pain, took paracetamol as the first medication. On the other hand, FM patients usually need a combination of different drugs, considering pain and psychiatric comorbidities. Here, we analyzed the following pharmacological subgroups in order to detect the percentage of expression of Mu+ NK cells in comparison with a specific therapy: antidepressants, myorelaxants, paracetamol, benzodiazepines, NSAIDs, nutritional supplement/none, and previous drug combinations. We found that, despite the different pharmacological medications, the percentage of Mu receptor on the NK cell surfaces was almost identical across the subgroups (mean 6) (Figure 5).

#### 3.3.3. Correlations between Patients’ Age with Mu+ NK Cells Percentage

**Figure 6:**
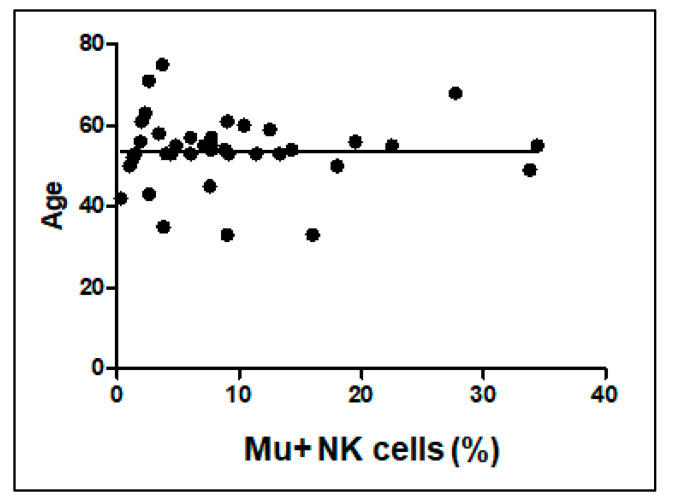
Age correlation with Mu+ NK cell percentages: no correlation was found. Linear regression: *p*-value 0.99; R-square 4 × 10^−6^.

We correlated Mu+ NK cell percentages with the ages of all the participants. We did not find any statistically significant correlations (Figure 6).

## 4. Discussion

The lack of specific diagnostic tools in chronic pain influences the choice of the right therapy for a single patient. Thus, in the last decades, scientists have focused their attention on the identification of pain biomarkers in order to obviate a possible misdiagnosis, delayed clinical results and useless therapies [7,26]. Considering pain as the main symptom of their pathologies, FM and OA subjects, as other chronic pain patients, need urgent answers about the classification of their pain in order to undertake the best-tailored therapy [27].

Recently, we hypothesized Mu+ B cell percentages of expression as candidate markers (MLM) of the pain intensity in OA and FM patients. Our previous data have already demonstrated the modulation of Mu+ B cells in these groups of people. In particular, Mu+ B cell percentages in pain-free subjects were significantly higher than the same percentage of expression in OA and FM patients. Moreover, Mu+ B cell percentages were related to the intensity of the pain. Patients with moderate/severe NRS pain scores showed lower Mu+ B cell percentages of expression than mild pain patients and pain-free people. We explained this result as a deficit of Mu opioid receptor reserve in pain patients. A low expression of MLM seems to correspond to a low threshold of pain perception and to a modified endogenous opioid analgesic activity. In the same study, we did not identify any association between the percentage of expression of Mu+ B cells and depression, anxiety, stress symptoms, pain acceptance, and illness representation [8].

The role of NK cells in the pain pathway has been analyzed in clinical studies [16], but the function of the Mu opioid receptor on their surfaces, as well as for B cells, remains to be understood. In the present study, we analyzed the role of the Mu opioid receptor on NK cells in the same patients in order to present NK cells as a new component of our eligible immune cell type setting implicated in the Mu opioid receptor reserve theory. Here, we observed that Mu+ NK cell percentages of expression were different between pain patients and pain-free subjects, as already seen for Mu+ B cells. Thus, we postulate a deficit of the Mu opioid receptor reserve also for NK cells, assuming again an altered endogenous opioid analgesic activity in FM and OA patients. Interestingly, we noticed that a low expression of Mu+ B cells corresponded to a low expression of Mu+ NK cells in the same single patients. Moreover, the Western blot analysis suggested a different functionality/activation of NK cells in pain patients compared to pain-free subjects.

These results lead us to deep down the relation between NK and B cells in chronic pain patients. In the last decades, the interplay between NK and B cells has been demonstrated [28]. Interestingly, in vitro experiments have shown that NK cells might directly activate B cells via cell–cell contact, without INF-gamma mediation and involving receptors and counterreceptors [29]. B cell activation due to a direct contact with NK cells is fast and could be considered as an autocrine process. This spontaneous condition could provide an early protective defense or, in pathological conditions, damaging mechanism. Interactions between activated B and NK cells are supported by several in vivo data [28]. In addition, human studies demonstrated that B and NK cells could cooperate in vitro, leading to conjugate the formation and stimulation of the interacting cells. Anyway, this mechanism is slightly understood [28]. NK cells are strictly involved in the innate immune system, able to spontaneously interact with either immune or non-immune cells. Several data suggested that NK cells could be related to the progression of the autoimmune pathology. Moreover, it has been shown that spontaneous interactions between B cells and autologous NK cells can be relevant for innate B cell reactions, leading to an initial benefit for the host or, in diverse conditions, to the progression of autoimmunity [28].

In 2009, Jennings and co-workers [30] presented NK cells’ involvement in the modulation of B cells’ ability to process and present antigens to T cells, underling the importance of this mechanism in immune memory generation.

Further studies are needed to understand the interplay between NK and B cells in chronic pain patients. Moreover, it could be interesting to extend the study of the Mu-Lympho-Marker to a larger cellular contest in order to analyze whether different cellular types could be involved in Mu opioid receptor modulation in chronic pain conditions. Further studies should also be directed to understand whether the modulation of B and NK cells presenting Mu could also be a common mechanism for other, different chronic pain conditions.

At the end, the utilization of a specific diagnostic biomarker could lead to an accurate recognition of the pain intensity, facilitating the prescription of the most suitable drug for the best patient management [27,31]. FM patients, for example, are usually overloaded by medical visits and pharmacological treatments because of psychiatric and psychological problems and/or biological and social environmental characteristics associated with their pathology [32]. Recent studies have also described a possible relationship between the systemic origin of FM and the gut microbiome, as well as for OA [33,34]. However, considering pain as the main symptom for both FM and OA, the use of pain biomarkers could avoid the patient’s waste of time in useless visits and possible side effects insurgence during the search for the right drug.

## 5. Conclusions

The complexity of pain pathophysiology strongly requires new therapeutic approaches in order to improve patients’ and caregivers’ quality of life. Pain biomarker availability could open the way for personalized therapies and more effective and safer rehabilitation programs [27,31]. Moreover, a peripheral marker utilization could reduce unnecessary suffering and costs for patients by limiting anxiety and stress due to a useless and excessive number of clinical exams. The peripheral localization of MLM could be relevant in the choice of opioid formulations that do not act at the central level, bypassing opioid drugs’ adverse effects.

In conclusion, the discovery of unique and objective diagnostic biomarkers seems to remain the most intriguing challenge in chronic pain medicine.

## Figures and Tables

**Table 1:** Patients’ characteristics.

Patients Characteristics	
Diagnosis	% of Patinets (*n* = 64)
Fibromyalgia (FM)	45% (*n*= 29)
Osteoatrithis (OA)	19% (*n* = 12)
No pain	36% (*n* = 23)
**Gender**	
Male	0% (*n* = 0)
Female	100% (*n* = 64)
**AGE (years)**	
20–29	0% (*n* = 0)
30–39	8% (*n* = 3)
40–49	10.5% (*n* = 4)
50–59	63% (*n* = 24)
60–69	13.5% (*n* = 5)
70–79	5% (*n* = 2)
mean 54	

## Data Availability

Not applicable.
